# Emergency CT Scans: Unveiling the Risks of Contrast-Associated Acute Kidney Injury

**DOI:** 10.3390/tomography10070080

**Published:** 2024-07-11

**Authors:** Omay Sorgun, Rezan Karaali, Cüneyt Arıkan, Efe Kanter, Güner Yurtsever

**Affiliations:** 1 Department of Emergency Medicine, Izmir Şehir Hospital, 35540 Izmir, Türkiye; 1912omay@gmail.com; 2 Department of Emergency Medicine, Izmir Democracy University, 35140 Izmir, Türkiye; rezantahtaci@hotmail.com; 3 Department of Emergency Medicine, School of Medicine, Dokuz Eylül University, 35340 Izmir, Türkiye; 4 Department of Emergency Medicine, Izmir Ataturk Research and Training Hospital, 35360 Izmir, Türkiye; efekanter@hotmail.com (E.K.); guner.yurtsever@gmail.com (G.Y.)

**Keywords:** contrast-associated acute kidney injury, emergency department, creatinine, Mehran score

## Abstract

Objectives: This study aimed to identify the incidence and risk factors for contrast-associated acute kidney injury nephropathy (CA-AKI) in patients undergoing contrast-enhanced computed tomography (CCT) in the emergency department. Materials and Methods: In this retrospective single-center study, patients aged 18 and older who visited the emergency department and underwent CCT between January and February 2022 were included. The Mehran score, calculated from patient data, was used to assess risk. CA-AKI development was determined by measuring serum creatinine (SCr) levels 48–72 h post-contrast administration. Results: The study included 532 patients, with a mean age of 57 ± 19 years; 53.2% were male. CA-AKI developed in 16% of cases, 5.82% required hemodialysis, and 7.9% died. The Mehran score was the only significant predictor of CA-AKI development. Patients with a Mehran score of 16 or higher had a 161-fold increased risk of developing CA-AKI compared to those with a score of 5 or lower. The model achieved a 91.3% correct classification rate. Logistic regression analysis showed that CA-AKI significantly increased mortality risk by 15.7 times. Conclusion: The Mehran score, originally developed for predicting CA-AKI risk post-coronary intervention, is also effective for predicting CA-AKI risk after CCT. While CA-AKI is a significant factor affecting mortality, it is not the sole cause of death (Nagelkerke R2 value 0.310).

## 1. Introduction

Contrast-associated acute kidney injury (CA-AKI) was first defined in 1954 and has been extensively studied since then [1]. The development of CA-AKI is intricate, and the precise underlying pathophysiological mechanism is still uncertain. CA-AKI occurrence is closely associated with increased blood viscosity, reperfusion injury, direct toxicity to renal tubule cells, and long-term vasoconstriction. In addition, there is a correlation between CA-AKI and abnormal physiological indicators and multiple conditions, such as elevated serum osmotic pressure and a Syntax score of II [2,3]. Contrast agents are used in patients undergoing imaging in the emergency department to assist in making diagnoses [4,5]. The use of contrast agents can lead to side effects such as CA-AKI [3,4]. CA-AKI is typically defined as an increase in the serum creatinine (SCr) level of more than 44.2 μmol/L (0.5 mg/dL), or a 25% increase compared to the baseline SCr level, occurring 48 h after exposure to iodine-based contrast [6]. It has been suggested that various factors may play a role in the pathogenesis of CA-AKI, including direct nephrotoxic effects of contrast agents, hemodynamic changes, oxidative stress, apoptosis, immune/inflammatory responses, and epigenetic regulation [5,7]. CA-AKI has been reported as the third most common cause of hospital-acquired acute kidney injury [8]. The incidence of CA-AKI in patients with initially normal kidney function is less than 5% [6]. The prevalence of CA-AKI in patients with pre-existing renal insufficiency is reported to be between 12% and 27% [5,7].

Physicians may hesitate to use contrast agents due to the risk of the patient developing CA-AKI. This situation can make it challenging to diagnose and provide effective treatment to patients. In the emergency department, it is not always possible to perform appropriate pre-contrast assessments, and prophylactic treatments may not be feasible for patients requiring urgent imaging, putting them at a higher risk for CA-AKI [9,10].

There is a need for indicators that can rapidly and effectively predict this risk in the emergency department. The Mehran score, developed to determine the risk of CA-AKI, was initially described in patients undergoing coronary angiography in 2004 [4]. This score accurately predicts the occurrence of contrast-induced nephropathy and categorizes patients based on their likelihood of experiencing negative clinical outcomes in the short and long term following primary percutaneous coronary intervention [11]. The risk score includes various factors such as hypotension, use of intra-aortic balloon pump, congestive heart failure, age, anemia, diabetes mellitus, contrast media volume, and estimated glomerular filtration rate (GFR). Each factor is assigned a specific number of points based on its severity or value. For example, hypotension is assigned 5 points if the systolic blood pressure is less than 80 mmHg for at least 1 h and requires inotropic support. Similarly, congestive heart failure is assigned 5 points if it is classified as class III/IV by the New York Heart Association or if there is a history of pulmonary edema. Age, anemia, diabetes mellitus, contrast media volume, and GFR are also assigned points based on specific criteria. Risk scores of 5 or less, 6 to 10, 11 to 15, and greater than 15 indicate risks for CIN of 7.5%, 14%, 26%, and 57%, respectively [11].

The aim of this study was to investigate factors that influence the development of CA-AKI in cases where computed tomography (CT) imaging is performed using contrast agents in the emergency department.

## 2. Materials and Methods

Our study was designed as a single-center, retrospective study. It was initiated based on the decision of the Izmir Katip Çelebi University Ethics Committee, dated 20 January 2022, with reference number 595. Patients who presented to our emergency department between 1 January and 28 February 2022 were retrospectively screened through the automated system. Only cases of patients aged 18 and over, who underwent contrast-enhanced computed tomography (CCT) imaging in the emergency department and had follow-up blood tests for serum creatinine (SCr) values taken 48–72 h after contrast administration, were included in the study.

Patients with pre-existing end-stage renal disease requiring dialysis, those who started or were scheduled to start hemodialysis or peritoneal dialysis within 45 days, individuals planned for kidney transplantation, those who received intravenous contrast within the last 14 days, pregnant women, and those within the first 48 h postpartum, trauma cases, individuals without follow-up blood SCr levels taken between 48–72 h, individuals with a history of nephrotoxic (NSAID, antibiotic, antineoplastic) drug use within the last 3 months, and those diagnosed with multiple myeloma were excluded from the study.

The age and gender of the cases were recorded. The presence of hypotension (SBP < 80 for ≥1 h requiring inotrope or balloon pump within 24 h of catheterization), the application of an intra-aortic balloon pump, the presence of heart failure and/or pulmonary edema, blood hematocrit (HCT) level, the presence of diabetes mellitus (DM), and the amount of contrast agent used were recorded from the patient files. Glomerular filtration rate (eGFR) was calculated using gender, age, and serum creatinine (SCr) level obtained from the blood test performed at admission to the emergency department.

The Mehran score was calculated based on these data. The obtained values were classified according to Mehran et al.’s classification, as follows: score ≤ 5 as low risk, 6–10 as moderate risk, 11–15 as high risk, and ≥16 as very high risk [1]. The development of contrast nephropathy was recorded based on the control SCr level obtained from the blood test taken 48–72 h after contrast administration. Patients with a serum SCr level showing an increase of more than 44.2 μmol/L (0.5 mg/dL) or a 25% increase compared to the baseline SCr level were classified as having contrast nephropathy (group 1), while those without these changes were classified as not having contrast nephropathy (group 2). The data obtained were compared between these two groups to determine the risk of developing contrast nephropathy.

The cases needing emergency dialysis were recorded. Comorbidities were classified into two groups according to the Charlson comorbidity index (CCI) as ≥5 and <5 [9]. The 30-day all-cause mortalities from initial admission to the emergency department were also recorded for the cases.

Our hospital’s emergency department utilizes a GE Revolution EVO 128 Slice tomography device (GE Healthcare, 3000 N. Grandview Blvd., Waukesha, WI, USA) for performing computed tomography scans. During the CT scan, a vial containing a solution for intravenous injection, which is equivalent to 300 mgI per milliliter and contains 647 mg of iohexol (OMNIPAQUE^®^ 300 mg I/mL IA, IV, GE Healthcare Ireland, Cork, Ireland or BİEMEXOL^®^ 300 mgI/mL IA, IV, İDOL İLAÇ DOLUM SAN. ve TİC. A.Ş., Istanbul, Türkiye), is used as the contrast agent.

### Statistical Analysis

The data obtained were analyzed using the IBM SPSS Statistics Version 20 software package. Descriptive statistics, frequency, and percentage distributions were calculated. For continuous variables, mean, standard deviation, minimum values, and maximum values were computed. The normality of continuous variables was assessed using the Kolmogorov–Smirnov and Shapiro–Wilk tests, and the appropriate statistical tests were selected based on the results. Chi-square tests were used to compare categorical variables between groups. For continuous variables, independent samples t-tests were employed for comparisons between two groups, while ANOVA tests were used for comparisons among more than two groups. To explore the potential effects of variables thought to be influential on contrast nephropathy, binary logistic regression analysis was conducted.

## 3. Results

During the study period, 1246 patients in our emergency department received contrast agents for CT scans. Among them, 532 cases that met the inclusion criteria and had sufficient data in their files were included in the study. The average age of the cases was found to be 57.31 ± 18.97 years. In total, 53.19% of the cases were male. It was observed that 47.36% of the cases were discharged from the emergency department, 15.78% developed contrast nephropathy, 5.82% underwent dialysis, and 7.9% died. When comparing the parameters considered in the study between cases with and without contrast nephropathy, statistically significant differences were found for age, HCT, SCr, eGFR, control SCr, systolic BP, Mehran score, DM, congestive heart failure (CHF), CCI, emergency HD, and mortality parameters (*p* < 0.005) (Table 1). For the statistically significant parameters, a two-step logistic regression analysis was performed. In the first step, all statistically significant variables were included in the model. The Mehran score and eGFR factors were found to be significant (*p* < 0.05). In the second step, it was concluded that the eGFR factor did not have an effect on the presence of contrast nephropathy, while the Mehran score variable was found to be effective. The probability of having CA-AKI increased 5.544 times in cases with a Mehran score between 6 and 11 compared to cases with a Mehran score ≤ 5, 67.414 times in cases with a Mehran score between 11 and 15, and 161.568 times in cases with a Mehran score of 16 or higher. The model’s correct classification rate is 91.3%, indicating its explanatory power (Table 2).

In our evaluation of mortality, statistically significant differences were found for age, SCr, eGFR, systolic BP, Mehran score, development of CA-AKI, DM, CHF, CCI, and emergency HD between deceased and surviving cases (*p* < 0.05) (Table 3). For the significant parameters, the first step in the logistic regression analysis revealed that the age, CA-AKI, and Mehran score variables were found to have an impact on mortality. In the second step, it was found that the age variable had a low effect on mortality, while the CA-AKI variable had a high level of influence. The odds of mortality in cases with CA-AKI development increased by 15.765 times. The model’s correct classification rate is 92.9%, indicating its explanatory power. The Nagelkerke R2 value is 0.310, indicating that the model explains the dependent variable to a low extent (31% rate) (Table 4).

## 4. Discussion

Contrast-associated acute kidney injury (CA-AKI) is a complicated and severe kidney injury that occurs due to the use of iodinated contrast agents, high-osmolar dyes, and direct toxicity to the kidney tubules [12]. CA-AKI is a prevalent cause of acute kidney injury in patients who are hospitalized. It lacks a definitive treatment, making prevention the most effective approach [13].

Proper hydration is crucial for patients who are given contrast, but there is no evidence that any medications or forced diuresis can significantly decrease the likelihood of contrast-induced nephropathy [14].

There are many studies on contrast nephropathy for pre-use prevention and post-use treatment [13,14,15]. However, there is still no clear prevention or treatment protocol for this undesirable condition. Fluid loading, especially before cardiac interventions [15], drugs such as N-acetylcysteine to support antioxidant mechanisms [16] in addition to fluid, carvedilol, metoprolol, adenosine [17,18], tibolone [19], dapagliflozin [20], and anti-inflammatory, anti-oxidant drugs like curcumin, berberine, and oxytocin [21,22,23] used experimentally in acute renal failure did not significantly change the response of the patients to the contrast agent.

Although the Mehran score is a scoring system developed to predict the likelihood of contrast nephropathy before coronary angiography, there is still no definitive scoring system in the literature. Miaoli et al. reported that a Mehran risk score of ≥17 was an independent predictor of persistent renal dysfunction (RD) [24]. Similarly, Wi et al. and Kakroo et al. reported that high risk (Mehran risk score 11–15) and very high risk (Mehran risk score ≥ 16) were independent predictors of persistent RD in patients with acute myocardial infarction undergoing percutaneous coronary intervention [25,26]. Sequeiros et al. also reported in a similar study that the performance of the score in predicting CA-AKI risk was high [8]. Our findings are consistent with these studies. In our regression analysis to determine the CA-AKI risk in the cases included in our study, we found that the Mehran score influenced the risk of CA-AKI. The Nagelkerke R2 value of our model was 0.310, indicating that it explained the likelihood of CA-AKI occurrence well. We found a specificity of 97.5% and a sensitivity of 57.8%. We observed that, as the Mehran score increased, the likelihood of developing CA-AKI also increased. Additionally, we found that age, HCT level, first SCr, eGFR, systolic BP, DM, CHF, and CCI were statistically different between cases with and without CA-AKI development. DM and a high level of glucose also worsen the probability of kidney damage. According to this knowledge, it is possible to evaluate the Mehran score with the level of blood glucose before interventions [27,28].

Studies evaluating cases undergoing CCT imaging have reported that age, DM, CHF, and eGFR are risk factors for CA-AKI [29,30,31]. In contrast to these studies, our regression analysis did not find these parameters as influential factors. However, the finding that the Mehran score, calculated using these parameters, was identified as an effective factor indicates that the Mehran score is a good predictor when deciding to perform CCT imaging in patients in the emergency department. In this study, although we found a CA-AKI development rate of 15.78% in the cases included, the rate of cases requiring emergency dialysis was 5.82%. Similarly, a study by Hinson et al. that compared cases undergoing CCT imaging with cases undergoing CT imaging did not find a significant difference in the rates of acute kidney injury development [32]. They emphasized the importance of considering potential mortality and morbidity due to life-threatening conditions when making the decision to administer contrast agents to patients [32]. The low rate of cases requiring dialysis in our study supports Hinson et al.’s viewpoint. When deciding on CCT imaging for patients, considering the risk–benefit ratio can be particularly helpful in managing critical patients.

CA-AKI is generally considered to be short-term and reversible; however, it is believed that, in some cases, it can lead to chronic kidney failure, prolonged hospitalization, and even death [33,34]. In a study by Levy et al., which evaluated cases undergoing CCT imaging, the mortality rate was found to be 34% in cases with CA-AKI. Even after excluding existing comorbid conditions, the mortality rate was reported to be 5.5%. They suggested that, although the primary cause of death might not be CA-AKI itself, conditions such as sepsis, bleeding, delirium, and respiratory failure may worsen due to CA-AKI, contributing to mortality [35]. Similarly, in a study by Mitchell et al., they reported an overall mortality rate of 7% and suggested that CA-AKI increased mortality related to other causes [33]. In our study, the overall mortality rate was 8.57%, and CA-AKI was detected in 66.7% of the deceased cases. According to our logistic regression analysis, CA-AKI was found to be a significant factor affecting mortality. However, we obtained a Nagelkerke R2 value of 0.310 and a sensitivity of 21.4%, indicating that the presence of CA-AKI alone accounted for a low level of mortality. Based on our findings, we can conclude that the presence of CA-AKI contributes to mortality to a low extent. However, as previously indicated in other studies, additional comorbid conditions should be considered when assessing mortality. According to our results, in the comparison of deceased and surviving cases, CCI, age, SCr, systolic BP, Mehran score, DM, and CHF were found to be statistically significant. However, in our regression analysis, none of these parameters were found to be significant factors influencing mortality individually. These findings suggest that mortality in patients undergoing contrast-enhanced CT imaging may be influenced by multiple factors, but the presence of CA-AKI may still increase the risk of mortality.

In a study by Heinrich et al. they evaluated the incidence of contrast-induced nephropathy (CIN) among patients receiving these contrast agents. On the other hand, they declare that iodixanol caused a significantly smaller increase in creatinine concentration compared to iohexol [36], which is mostly used in the hospital in which this study was performed.

Today, angiographies are performed using low contrast [37]. In the same way, in our opinion, it is clear that targeted imaging techniques with low-dose contrast should be switched to targeted imaging techniques with a good radiology technician, taking into account details such as weight, height, gender, etc. when performing contrast-enhanced CT, as Omigbodun et al. described [38].

In a study conducted by Kane et al. they describe that patients with chronic kidney disease who undergo coronary angiography are at a lower risk of developing contrast-induced nephropathy when using ultra-low contrast volumes [39]. Moreover, they found that the volume of contrast used in patients who developed contrast-induced nephropathy (CIN) was higher (45 ± 18 mL) compared to those who did not (31 ± 18 mL). In the literature, a maximum dose of 100–120 mL of contrast medium is recommended for abdominal CT [40], while 1–2 mL/kg is recommended for CT of other parts of the body [41]. In a study by Araki et al. they found that abdominopelvic computed tomography using low voltage (80-kVp) enables a 60% reduction in the dose of contrast agent for patients who are at risk of developing contrast-induced nephropathy [42]. On the other hand, the utilization of low-kVp protocols has the effect of enhancing vascular visibility, decreasing the amount of contrast media required, and, when implemented with the MBIR algorithm, substantially reducing image noise [43]. In our study, although we thought that CA-AKI risk would be less with low contrast doses and professional CT use, dose ranges were not calculated, because there was no system to record the volumes. Future studies will clarify this situation.

In emergency scenarios, there may be limited time to collect all the data needed for an accurate Mehran score. Rapid assessment tools and streamlined protocols are necessary to integrate this into emergency care effectively. The Mehran score was originally validated for PCI, so its direct application to CT scans might require some adaptation or additional validation to ensure accuracy and relevance. Emergency situations can sometimes result in incomplete or inaccurate patient histories, which can impact calculation of the Mehran score. In emergencies, the need for rapid intervention may outweigh the risk of CA-AKI, necessitating a careful balance between the urgency of the procedure and the risk assessment provided by the Mehran score.

## 5. Conclusions

In conclusion, this study demonstrated that the Mehran score may be a good predictor of CA-ACI risk in patients undergoing emergency CT. Although the Mehran score has not been designed as a predictive tool for emergency departments, we think that this score can be used not only for PCI but also for contrast-enhanced CT in the emergency department and will guide physicians correctly in the rapid decision-making phase, which is the nature of the emergency department.

## 6. Limitations

The study has many limitations. Firstly, the study is a retrospective study. The lack of a control group due to the retrospective nature of the study is one of the weaknesses of the study. Moreover, as this study is retrospective we could not obtain all the information of patients, which is important for Mehran score calculation. We did not compare different fluids as iohexol and iodixinol used as contrast. On the other hand, as the pathology for which the contrast agent is administered may also be a determining factor, we did not observe other pathologies.

## Figures and Tables

**Table 1:** General characteristics of the parameters discussed in the study and comparison of the parameters between patients with and without contrast-induced nephropathy.

Variables		CA-AKI (*n* = 84)	No CA-AKI (*n* = 448)	*p*
		Mean ± SD (Min–Max)	Mean ± SD (Min–Max)	
Amount of contrast/mL		52.38 ± 12.95 (30–60)	53.78 ± 11.8 (5–60)	0.36
Age/year		68.64 ± 16.82 (26–99)	55.19 ± 18.61 (18–99)	<0.001
HCT		36.15 ± 7.68 (19.2–55.5)	36.6 ± 6.6 (13.5–60.6)	<0.001
1. Creatinine—mg/dL		1.43 ± 0.87 (0.51–5.86)	1.04 ± 0.45 (0.47–7.3)	<0.001
eGFR		56 ± 26.4 (9–118)	81.21 ± 21.76 (7–133)	<0.001
2. Creatinine—mg/dL		3.25 ± 1.43 (1.24–8.06)	1.18 ± 2.77 (0.37–42)	<0.001
Systolic BP/mmHg		119.35 ± 25.81 (69–220)	126.01 ± 17.79 (85–210)	<0.001
**Variables**		***n* (%)**	***n* (%)**	***p***
Gender	Male	45 (15.9)	238 (84.1)	0.52
	Female	39 (15.7)	210 (84.3)	
DM	None	43 (10.2)	379 (89.8)	<0.001
	Exist	41 (37.3)	69 (62.7)	
CHF	None	48 (10.3)	420 (89.7)	<0.001
	Exist	36 (56.3)	28 (43.8)	
CCI	≤5	38 (8.8)	392 (91.2)	<0.001
	>5	45 (44.6)	56 (55.4)	
Urgent HD	No	55 (11.0)	446 (89.0)	<0.001
	Yes	29 (93.5)	2 (6.5)	
Clinical Decision	Discharged	13 (5.2)	239 (94.8)	<0.001
	Ward	36 (18.2)	162 (81.8)	
	ICU	35 (42.7)	47 (57.3)	
Mortality	Survived	56 (11.4)	434 (88.6)	<0.001
	Deceased	28 (66.7)	14 (33.3)	
Mehran Score	≤5	14 (3.8)	353 (96.2)	<0.001
	6–10	21 (20.0)	84 (80.0)	
	11–15	38 (79.2)	10 (20.8)	
	≥16	11 (91.7)	1 (8.3)	

CA-AKI: contrast-associated acute kidney injury, HCT: hematocrit, eGFR: glomerular filtration ratio, BP: blood pressure, DM: diabetes mellitus, CHF: congestive heart failure, CCI: Charlson comorbidity index, HD: hemodialysis, ICU: intensive care unit.

**Table 2:** Determination of CA-AKI risk factors by logistic regression analysis of parameters with significant difference between patients with and without CA-AKI.

Variables in the Equation									
		B	SE	Wald	df	Sig.	Exp (B)	95% CI for EXP (B)	
								Lower	Upper
Step 1 ^a^	Age	−0.002	0.011	0.019	1	0.889	0.998	0.976	1.021
	HCT	0.066	0.08	0.672	1	0.412	1.068	0.913	1.25
	Creatinine	−0.533	0.302	3.112	1	0.078	0.587	0.324	1.061
	eGFR	−0.02	0.009	4.669	1	0.031	0.981	0.963	0.998
	Systolic BP	−0.001	0.01	0.014	1	0.907	0.999	0.979	1.019
	DM (1)	0.269	0.384	0.492	1	0.483	1.309	0.617	2.78
	CHF (1)	0.686	0.469	2.145	1	0.143	1.986	0.793	4.976
	CCI (1)	−0.095	0.438	0.047	1	0.827	0.909	0.385	2.144
	Mehran score			30.098	3	0			
	Mehran score (1)	1.524	0.502	9.207	1	0.002	4.589	1.715	12.277
	Mehran score (2)	3.914	0.732	28.565	1	0	50.079	11.922	210.354
	Mehran score (3)	4.847	1.373	12.458	1	0	127.402	8.633	1880.122
	Constant	−1.424	1.956	0.53	1	0.467	0.241		
**Variables in the Equation**									
		**B**	**S.E.**	**Wald**	**df**	**Sig.**	**Exp(B)**	**95% CI for EXP (B)**	
								**Lower**	**Upper**
Step 2 ^a^	eGFR	−0.011	0.007	2.359	1	0.125	0.989	0.975	1.003
	Mehran score			78.878	3	0			
	Mehran score (1)	1.713	0.376	20.767	1	0	5.544	2.654	11.581
	Mehran score (2)	4.211	0.493	72.974	1	0	67.414	25.655	177.149
	Mehran score (3)	5.085	1.129	20.281	1	0	161.568	17.67	1477.301
	Constant	−2.301	0.648	12.604	1	0	0.1		

Step 1 ^a^. Variable(s) entered on step 1: age, HCT, CREATININE, GFR, SISTOLIC BP, DM, CHF, CCI, MEHRAN SCORE. Step 2 ^a^. Variable(s) entered on step 1: eGFR, Mehran score. CA-AKI: contrast-associated acute kidney injury, HCT: hematocrit, eGFR: glomerular filtration ratio, BP: blood pressure, DM: diabetes mellitus, CHF: congestive heart failure, CCI: Charlson comorbidity index.

**Table 3:** Comparison of parameters between deceased and surviving patients.

Mortality Table				
Variables		None (*n* = 490)	Yes (*n* = 42)	*p*
		Mean ± SD (Min–Max)	Mean ± SD (Min–Max)	
Amount of contrast/mL		53.72 ± 11.86 (5–60)	51.67 ± 13.42 (30–60)	0.31
Age/year		56.16 ± 18.68 (18–99)	70.74 ± 17.24 (26–99)	<0.001
HCT		39.21 ± 6.78 (13.5–60.6)	37.17 ± 7.83 (22.2–54.6)	0.11
1. Creatinine—mg/dL		1.07 ± 0.48 (0.47–5.86)	1.42 ± 1.07 (0.51–7.3)	<0.001
eGFR		78.23 ± 23.58 (9–133)	65.6 ± 29.8 (7–114)	0.01
2. Creatinine—mg/dL		1.39 ± 2.74 (0.37–42)	2.84 ± 1.82 (0.59–8.06)	<0.001
Systolic BP/mmHg		125.29 ± 19.44 (69–220)	121.05 ± 18.69 (85–167)	0.33
**Variables**		***n* (%)**	***n* (%)**	***p***
Gender	Male	258 (91.2)	25 (8.8)	0.39
	Female	232 (93.2)	17 (6.8)	
Contrast nephropathy	No	434 (96.9)	14 (3.1)	<0.001
	Yes	56 (66.7)	28 (33.3)	
DM	None	396 (93.8)	26 (6.2)	<0.001
	Exist	94 (85.5)	16 (14.59)	
CHF	None	437 (93.4)	31 (6.6)	0.01
	Exist	53 (82.8)	11 (17.29)	
CCI	≤5	409 (95.1)	21 (4.9)	<0.001
	>5	80 (79.2)	21 (20.8)	
Mehran score	≤5	354 (96.5)	13 (3.5)	<0.001
	6–10	90 (85.7)	15 (14.3)	
	11–15	38 (79.2)	10 (20.8)	
	≥16	8 (66.7)	4 (33.3)	
Urgent HD	No	477 (95.2)	24 (4.8)	<0.001
	Yes	13 (41.9)	18 (58.1)	

CA-AKI: contrast-associated acute kidney injury, HCT: hematocrit, eGFR: glomerular filtration ratio, BP: blood pressure, DM: diabetes mellitus, CHF: congestive heart failure, CCI: Charlson comorbidity index, HD: hemodialysis.

**Table 4:** Determination of risk factors affecting mortality by logistic regression analysis of statistically significant parameters between deceased and surviving patients.

		B	SE	Wald	df	Sig.	Exp (B)	95% CI for EXP(B)	
								Lower	Upper
Step 2 ^a^	Age	0.031	0.012	6.847	1	0.009	1.031	1.008	1.056
	Contrast nephropathy (1)	2.758	0.459	36.128	1	0.000	15.765	6.414	38.746
	Mehran score			4.496	3	0.213			
	Mehran score (1)	0.476	0.472	1.016	1	0.313	1.610	0.638	4.060
	Mehran score (2)	−0.627	0.602	1.087	1	0.297	0.534	0.164	1.737
	Mehran score (3)	−0.337	0.811	0.172	1	0.678	0.714	0.146	3.498
	Constant	−5.423	0.794	46.606	1	0.000	0.004		

Step 2 ^a^ Variable(s) entered on step 1: Age, Contrast nephropathy, Mehran score.

## Data Availability

All the data for this study are presented in the published article. Any further details are available from the corresponding author (Cüneyt Arikan, dr.carikan@gmail.com or cuneyt.arikan@deu.edu.tr) upon reasonable request.
